# Predicting CO_2_ emissions and strength of FA-SF geopolymer mortar: An ANN approach for sustainable construction

**DOI:** 10.1371/journal.pone.0336654

**Published:** 2026-04-03

**Authors:** Jahanzeb Javed, Muhammad Usman Siddiq, Muhammad Akbar, Muhammad Usama Aslam, Abdullah Naveed Sadiq, Mahmoud Elkady, Ahmed M. Yosri

**Affiliations:** 1 Belfast School of Architecture and the Built Environment, Ulster University, Belfast, Northern Ireland, United Kingdom; 2 Civil and Building Services Engineering Division, School of Built Environment and Architecture, London South Bank University, London, United Kingdom; 3 School of Naval Architecture & Ocean Engineering, Jiangsu University of Science and Technology, Zhenjiang, Jiangsu, China; 4 Department of Civil Engineering, Northern Border University, Arar, Saudi Arabia; 5 Department of Disaster Mitigation for Structures, Tongji University, Shanghai, China; 6 School of Civil Engineering, Faculty of Engineering, Universiti Teknologi Malaysia, Skudai, Johor Bahru, Malaysia; 7 Department of Civil Engineering, College of Engineering, Jouf University, Sakaka, Saudi Arabia; SASTRA Deemed University, INDIA

## Abstract

The construction industry is facing increasing pressure to reduce the CO_2_ emissions from conventional cement manufacturing. Geopolymer concrete, utilizing fly ash (FA) and silica fume (SF) as precursors, presents a promising sustainable alternative. This study investigates the mechanical properties and environmental performance of FA- and SF-based GPC through a comprehensive experimental campaign and predictive modeling. This study conducted destructive (DT) and non-destructive testing (ultrasonic pulse velocity [UPV] measurement) of the fresh and hardened properties of GPC, focusing on the effect of steel fiber reinforcement on flexural strength and resilience. Based on life cycle assessment, artificial neural networks (ANNs) were used to predict both mechanical properties and CO_2_ emissions of GPC mixes. The ANN models demonstrated accurate predictions (R² = 0.96–0.99) and exhibited low errors. A SHAP-based sensitivity analysis identified key input parameters influencing ANN predictions. From the results, it can be concluded that optimized GPC mixes containing FA and SF can achieve gains of 20–25% in early-age compressive strength and a 15–20% reduction in CO_2_ emissions compared to OPC. The findings of this research are significant for designing long-life, high-performance GPC mixes. The resulting ANN-based predictive tool provides a practical approach for engineers and materials scientists to design GPC mixes that meet specific performance requirements and sustainability objectives, thereby promoting the development of low-carbon construction materials.

## 1. Introduction

The demand for Portland cement has grown dramatically to meet the needs of modern construction projects. However, the cement industry is a significant contributor to global CO_2_ emissions, accounting for nearly 8% of worldwide emissions. Cement production, particularly through the calcination process in clinker manufacturing, emits substantial CO_2_, exacerbating environmental concerns. In response to these challenges, governments and regulatory bodies worldwide have implemented stringent carbon-emission regulations to reduce the environmental impact of construction materials. Consequently, extensive research has been conducted to identify alternative materials that can replace conventional cement, particularly to reduce its carbon footprint [1–3].

**Fig 1:**
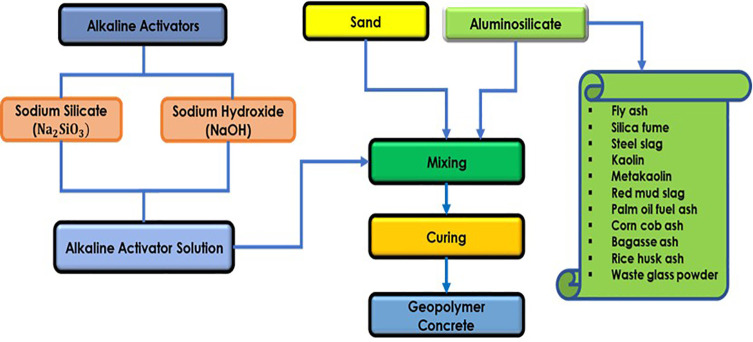
Manufacturing process of geopolymer concrete.

One such promising alternative is geopolymer concrete composite (GPC), which was first introduced by Davidovits in the 1970s as a novel technology for reducing CO_2_ emissions from construction materials [4,5]. Geopolymers are a class of inorganic, aluminosilicate-based materials that, when activated by an alkaline solution, can serve as effective binders for concrete, offering an opportunity to entirely replace conventional cement in construction. Geopolymer concrete has been shown to exhibit excellent mechanical strength and durability under extreme temperature conditions, making it a more sustainable and cost-effective solution compared to ordinary Portland cement (OPC) [5–7]. The low CO_2_ emissions associated with GPC arise from its reliance on industrial waste products, such as fly ash (FA) and silica fume (SF), rather than energy-intensive clinker-based cement [8–10]. Fig 1 shows the schematic diagram of the manufacturing process of geopolymer concrete.

The binder in GPC is formed by the reaction of alkaline solutions with aluminosilicate-rich precursors such as FA and SF. Fly ash, a coal by-product, is rich in aluminum oxide (Al₂O₃) and silicon dioxide (SiO₂), making it an economical and readily available source material for geopolymer production. Additionally, the production of FA-based geopolymers results in structural linkages, such as Si-O-Al-O-Si, via polymerization reactions, creating a highly durable, rigid matrix [11,12]. The incorporation of waste materials like recycled glass powder (GP), blast furnace slag (BFS), and agricultural by-products such as rice husk ash (RHA) and sugarcane bagasse ash (SCBA), further enhances the sustainability of GPC, diverting industrial waste from landfills while reducing reliance on OPC [13].

While the environmental and mechanical advantages of GPC have been well established, several research gaps remain, as presented in Table 1. Firstly, while FA has been extensively studied as a base binder in geopolymer concrete, there has been limited research on the combined use of FA and SF [14–16]. SF, another industrial by-product, has been shown to enhance the mechanical properties of concrete when combined with other binders, yet its role in geopolymer systems, particularly in optimizing CO_2_ emissions and mechanical strength, has not been thoroughly explored.

**Table 1:** Summary of recent literature studies on LCS of FA and SF-based GPC.

Reference	material	Focus	Environmental Impact (LCA)	Evaluated properties	Key findings	Limitations
Bajpai et al., 2020 [14]	FA and SF	LCA of geopolymer concretes vs conventional cement concrete	Lower global warming potential compared to conventional cement concrete	Compressive strength only	Reported lowest environmental impact; cost savings of 10.87%–17.77%	It did not include long-term durability, lacked detailed experimental tests, and ANNs modelling
Zar et al., 2023 [15]	FA, SF; GGBFS and recycled coarse aggregates	Techno-environmental feasibility of using FA, GGBS, and SF in GPC with 100% RA	LCA shows a 50–60% reduction in environmental impacts compared to traditional OPC concrete with NA.	Compressive, flexural, and splitting tensile strength, microstructural properties (SEM, XRD)	Compressive strength is 18–34% higher than OPC concrete.	Limited durability assessment and modelling
Onyelowe et al., 2022 [16]	FA and SF	Multi-objective study on compressive strength and environmental impact of FA and SF concrete mixes	Portland cement accounts for 90% of GWP; reduction of cement dosage via FA/SF reduces environmental impacts	Compressive strength, environmental impact (GWP, TAP), toxicity indicators	ANN model showed the highest prediction accuracy (performance index of 0.986) for compressive strength; FA and SF reduce CO₂ emissions and environmental impacts due to lower cement usage	Focus on compressive strength; lacks detailed analysis of other mechanical properties (e.g., durability). Limited to specific mix designs. Economic feasibility not addressed; sensitivity analysis absent
Kumar et al., 2021 [17]	FA and SF	Impact of FA and SF individually and combined as a binary cementitious material (BCM) on roller compacted concrete	Embodied carbon is reduced with increasing replacement of SF and FA	Compressive strength, splitting tensile strength, and water absorption	Compressive and tensile strength increased while Water absorption decreased with SF and FA.	Durability tests were not thoroughly discussed. Economic feasibility is not addressed; sensitivity analysis and ANN modelling are absent.

Secondly, while experimental studies have investigated the mechanical properties of GPC, predictive modeling tools that can optimize both performance and sustainability outcomes are lacking. Recent advancements in artificial intelligence (AI) and advanced machine learning (ML), such as artificial neural networks (ANN), offer a powerful approach for predicting material behavior using experimental datasets [18,19]. ANN has been successfully applied to predict the strength of traditional concrete [20,21], but its application to GPC, particularly systems based on FA and SF, remains underexplored. Recent studies, such as Nguyen et al. [20] and Rehman et al. [21], have shown that ensemble learning algorithms can enhance the prediction accuracy of concrete’s mechanical properties. Furthermore, the ability of ANNs to simultaneously predict mechanical properties and CO_2_ emissions in geopolymer materials has not been fully investigated [22,23]. Our study contributes to this growing body of work by employing an artificial neural network to optimize both the mechanical strength and sustainability of geopolymer concrete.

In addition to these gaps, few studies [14–16] have comprehensively examined the impact of multiple variables, including FA/SF ratios, curing conditions, and activator concentrations, on both mechanical performance and environmental metrics. There is a critical need for more in-depth research that explores the interaction of these variables to optimize GPC mixes for both sustainability and cost-effectiveness [24,25]. To date, no study has conducted detailed material characterization and CO_2_ emission prediction using ANN, based on interaction profiling of experimental datasets for FA- and SF-based geopolymer concrete.

Therefore, this work aims to explore the mechanical and environmental performance of FA/SF sustainable geopolymer mortar, including compressive, flexural, splitting, and tensile strengths, ultrasonic pulse velocity, and CO_2_ emissions. This study seeks to quantify the synergistic effects of FA and SF on these properties, then build ANN models that estimate mechanical strength and environmental effects with at least 90% accuracy. The distinctive feature of this study is that it integrates a large experimental dataset from DT and NDT tests to optimize both mechanical and environmental performance. This not only increases the credibility of the results but also enables examination of how material composition influences mechanical properties and sustainability. The two-sided focus on optimizing both mechanical properties and CO_2_ emissions sets this research apart as a true breakthrough in sustainable building materials. The results are directly aligned with the pressing needs of the construction industry to reduce carbon emissions and enhance material efficiency. By providing engineers and material scientists with an ANN-based predictive tool, this research facilitates the development of more efficient mix designs that can lead to reduced material waste and faster adoption of GPC in real-world applications. Ultimately, these study findings will advance sustainable construction practices, supporting industry goals for low-carbon development while maintaining high-performance standards.

While recent studies have applied ANN to predict the compressive strength of geopolymer concrete [18,19,25], significant gaps remain in integrated sustainability-performance modeling. Unlike previous works that focused solely on mechanical properties [21,26,27], this study presents a dual-objective ANN framework capable of simultaneously predicting mechanical strength (CS, STS, UPV) and environmental impact (CO₂ emissions) with >96% accuracy. Furthermore, incorporating SHAP-based sensitivity analysis enables interpretable optimization of mix designs for sustainable construction, a capability absent in existing ‘black-box’ ANN applications to GPC [20,23]. The experimental validation, through steel fiber corrosion resistance and residual flexural strength testing, provides additional practical insights for structural applications that are rarely addressed in predictive modeling studies.

## 2. Materials and methodology

The research methodology of this study comprises two phases: experimental testing and prediction analysis using an ANN model. Twelve input variables were tested to examine their effects on the fresh and mechanical strength, and CO₂ emissions, of FA- and SF-based GPC.

### 2.1 Specifications of raw materials

In this study, industrial byproducts, i.e., fly ash (FA) and silica fume (SF), were used as the primary materials for the production of geopolymer concrete. Both materials were procured from the same supplier to eliminate variability in results caused by differences in source ingredients. FA was partially replaced by SF at varying percentages to enhance performance across different curing ages and temperature conditions.

A predefined ratio of sodium hydroxide (NaOH) and water glass (Na₂SiO₃) at 1:2.5 was used to produce an alkali-activated solution. The sand used for the mortar was locally sourced and had particle sizes smaller than 4.75 mm. Additionally, a superplasticizer, Naphthalene, was added to improve the workability of the fresh polymer composite by reducing the viscosity caused by the reaction between the alkaline activators. Table 2 shows the chemical makeup of ordinary cement, FA, and SF content.

**Table 2:** Chemical composition of SF, FA, and OPC.

Compound	SF (%)	FA (%)	OPC (%)
${𝐒𝐢𝐎}_{2}$	91.90	55.28	15.0
${𝐀𝐥}_{2}{𝐎}_{3}$	0.71	26.71	2.78
${𝐅𝐞}_{2}{𝐎}_{3}$	2.54	6.65	2.72
𝐂𝐚𝐎	0.31	2.34	71.06
𝐌𝐠𝐎	1.14	0.81	1.38
${𝐒𝐎}_{3}$	0.45	0.47	4.56
${\;𝐊}_{2}𝐎$	1.53	N/A	1.21
${𝐓𝐢𝐎}_{2}$	0.01	1.89	N/A
${𝐏}_{2}{𝐎}_{5}$	0.63	1.92	N/A
𝐂𝐥	0.28	N/A	0.08
𝐌𝐧𝐎	0.26	N/A	N/A

### 2.2 Design of mix proportions for GPC

The process of determining the GPC mix design proportions differs from that for conventional concrete. In conventional mortar, the water-to-OPC ratio is measured first, then fine aggregate is added. In contrast, geopolymer mortar mix design includes more variables, such as source ingredients, alkaline activator solution, NaOH/Na_2_SiO_3_ ratio, curing conditions, and NaOH molarity, making strength balancing challenging.

This study used seventy-six design mixes to investigate the influence of various factors on the fresh and hardened concrete properties of FA and SF-based GPC mortar cured at 27 °C and 40 °C. Various FA and SF-based mortar mixes were evaluated, with different SF content, selected based on previous studies that showed improved mechanical performance and sustainability. These ratios were chosen for cost-effectiveness, mechanical strength, and environmental impact while optimizing carbon emissions reduction. All mixtures were based on a concrete volume of one m³, with each blend containing 545 kg/m³ of binder. The mass proportions of FA and SF were determined accordingly, as presented in Table 3.

**Table 3:** Mix design proportions for FA-SF-based GPC.

Mix ID	Mix proportion (unit, kg/m^3^)						
	OPC	FA	Silica Fume	NaOH	Na_2_SiO_3_	H_2_O	SP
**Control**	545	0	0	0	0	125	0
**100FA-0SF-0PC**	0	545	0	41	103	0	9
**90FA-10SF-0PC**	0	490	54.5	41	103	0	9
**60FA-40SF-0PC**	0	327	218	41	103	0	9

### 2.3 Development and preparation of GPC

A drum mixer was used to prepare all mixes, with aggregates’ surface dried according to ASTM standards just before blending. The alkaline activators NaOH and Na_2_SiO_3_ were mixed in predefined ratios one day prior to blending to create the solution. For all blends, the mixing technique and duration remained constant. The mixer was initially filled with dry materials, starting with sand, followed by the binder ingredients. The alkaline activator solution is added after 2 minutes of dry mixing. If needed, a superplasticizer was added, and the mix was blended for an additional 3 minutes for homogeneity. The molds were then filled with the geopolymer paste, and a mechanical vibrator was used to compact the specimens. After 24 hours, the samples were carefully demolded and placed at 25°C and 40°C under 75% relative humidity until testing.

### 2.4 Testing methods

**Fig 2:**
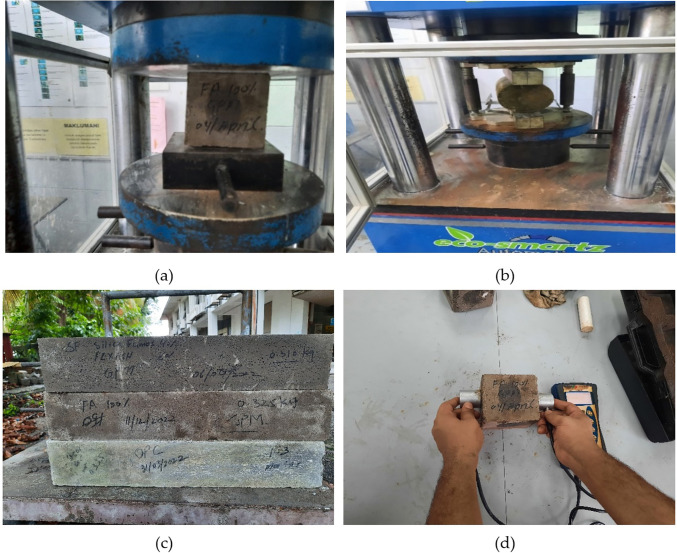
Experimental testing setup for FA and SF-based GP-Mortar: (a) Compressive strength; (b) Splitting Tensile Strength; (c) Residual Flexural; (d) Ultrasonic pulse velocity (UPV).

The slump flow test, conducted in accordance with ASTM standards, assessed the workability of freshly prepared FA-SF-based GPC blends. Hardened strength properties, such as compressive strength (CS), splitting tensile strength (STS), and ultrasonic pulse velocity (UPV), were evaluated to estimate GPC performance, as shown in Fig 2. CS testing was performed on cube specimens measuring 100 x 100 x 100 mm at 7, 14, and 28 days, in accordance with BS standards. STS testing was conducted on 100 x 200 mm cylinders at the same ages specified in ASTM standards. Residual flexural strength (RFS) tests were conducted on 100x100x400 mm prisms with 10 mm diameter reinforced steel bars after 28 days, per ASTM standards for PCC. UPV assessments were also performed on all concrete mix designs to evaluate the effects of multiple variables on GPC mortar performance.

### 2.5 Gravimetric weight loss measurement for steel fiber reinforced GPC

**Fig 3:**
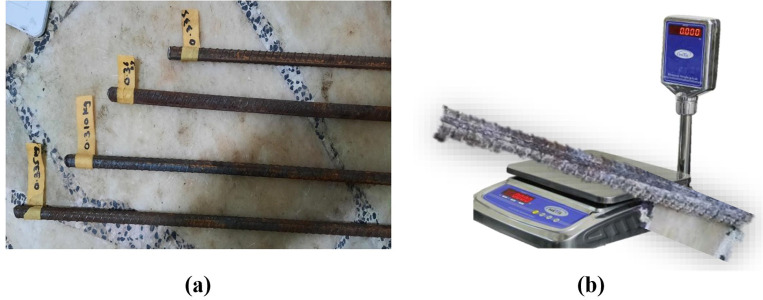
(a) 10 mm steel bar and (b) Weight loss of the steel bar to evaluate corrosion.

Gravimetric weight-loss measurement (GWL) is a fundamental destructive method for estimating corrosion by measuring the weight loss of a steel bar before and after corrosion. It is used to estimate corrosion in steel bridges. In the meantime, due to its excellent accuracy and ease of use, the GWL assessment in a controlled laboratory setting has been dubbed the gold standard test for various corroded steel buildings [28], which represents the average weight loss over the length considered, and was measured as the loss in weight over the original length portion of the rebar. Fig 3 shows the 10 mm steel bar and the weight balance to evaluate corrosion.

### 2.6 CO_2_ emissions and cost analysis calculations

The computation of the CO_2_ footprint for each FA- and SF-based geopolymer composite considers emissions associated with the extraction and manufacture of materials, as well as the curing temperature. This is calculated using the heuristic formula given in Eq. 1, defined by Torres et al. [29]:


$${CO}_{\text{2}}\;footprint\;\left(\frac{kg}{m^{\text{3}}}\right)=\sum_{i}\;w_{i}\cdot m_{i}+(\text{0}.\text{6}\text{4}\text{2}T-\text{1}\text{6}.\text{0}\text{4}\text{2})\cdot t$$ (1)

where: $w_{i}$ = CO_2_ footprint (kg) to production one m^3^ of the mixed component “*i”*. mi = mass of a component “*i”* in *kg/m*^*3*^ of fresh blend, “*T”* = curing temperature (◦C), *t* = curing age (days). The first part of the equation aggregates the CO_2_ footprint of each constituent by its mass. The second part incorporates the curing duration “*t*” into the computation only if the curing temperature (T) exceeds 25 °C; otherwise, it is set to 0.

Sustainability and economic analysis focused on one m³ of each alkali-activated geopolymer concrete composite (GPC) paste, specifically the control mix, 100FA-0SF-0PC, 90FA-10SF-0PC, and 60FA-40SF-0PC. Two indices were introduced for evaluating material properties: the CO_2_ emission index (CEI) and the cost index (CI) given in eq’s 2 & 3. The CEI normalizes total CO_2_ emissions per m³ of PCC against its compressive strength (CS), with lower values indicating better sustainability. Conversely, the CI measures economic return by evaluating the cost per m³, normalized by compressive strength, with smaller values signifying higher economic return.


$$CEI=\frac{{CO}_{\text{2}}\;emission\;\left(kg{CO}_{\text{2}}/{\;m}^{\text{3}}\;\right)}{f_{c}}$$ (2)

$$CI=\frac{Cost(\$/{\;m}^{\text{3}}\;)}{f_{c}}$$ (3)

where, $f_{c}$ = compressive strength at 28 days, *CEI* = CO_2_ emission index (unit, kgCO_2_/ m^3^) and *CI* = cost index (unit, $/m^3^).

### 2.7 Artificial Neural Network (ANNs) modelling

**Fig 4:**
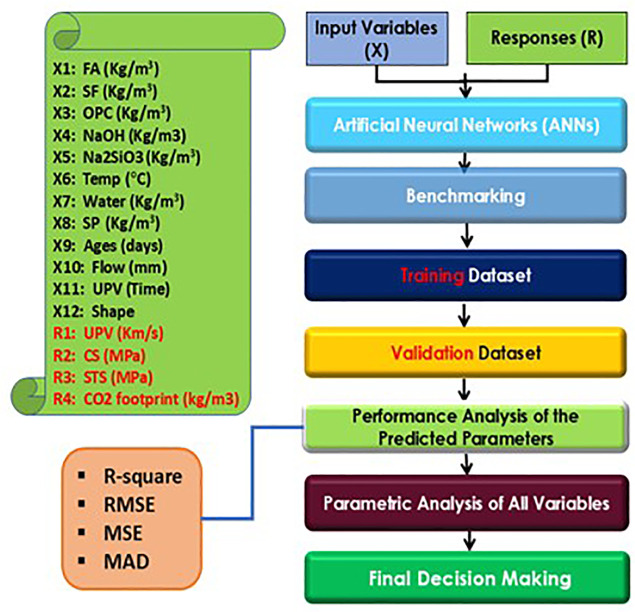
A comprehensive schematic research methodology was adopted for the ANN model.

Artificial Neural Networks, or ANNs, are computational models designed to mimic biological systems, consisting of three layers: input, hidden, and output [30–33]. This study employed a backpropagation method with a random holdback approach to train and validate data sets for predicting CO_2_ emissions and mechanical parameters of geopolymer concrete. The ANN’s weights were adjusted to minimize root mean squared error (RMSE), aiming for an R-squared value close to 1 to ensure model validity [34]. Input variables were normalized to the range 0–1 [35,36], facilitating compatibility with the sigmoid activation function and thereby enhancing the model’s performance. Additionally, preprocessing steps, such as feature scaling, were implemented in this study to address discrepancies in variable magnitudes, thereby further improving the ANN’s predictive capabilities. Overall, this methodology demonstrates the effectiveness of ANNs in modeling complex interactions in construction materials. Fig 4 shows the research methodology for the predicted model.

#### 2.7.1 Dataset specifications and preprocessing.

The experimental dataset comprised 76 distinct mix designs derived from destructive (DT) and non-destructive (NDT) testing protocols. The dataset was split into 50 training samples (65.8%) and 26 validation samples (34.2%), selected using a stratified random holdout approach to ensure a representative distribution across all input variable ranges.

Data preprocessing included: (1) outlier detection using the Interquartile Range (IQR) method (no outliers removed as all values fell within 1.5 × IQR bounds); (2) normalization of input variables to [0,1] range using min-max scaling (Equation 4) to ensure compatibility with sigmoid activation functions; and (3) feature engineering for categorical variables (specimen shape encoded as: cube = 1, cylinder = 2, beam = 3).


$$X_{\text{norm }}=\frac{X-X_{min}}{X_{max}-X_{min}}$$ (4)

where X norm is the normalized value, X is the original value, and X_min_/X_max_ are the minimum and maximum values in the dataset. Input variables included: FA (0–545 kg/m³), SF (0–218 kg/m³), OPC (0–545 kg/m³), NaOH (41 kg/m³ constant), Na₂SiO₃ (103 kg/m³ constant), curing temperature (27–40°C), water (0–125 kg/m³), superplasticizer (0–16.35 kg/m³), curing age (7–28 days), flow diameter (182–222 mm), UPV time (variable), and specimen shape (1–3).

The application of an ANN to this limited dataset (76 mixes) is justified by the structured, physics-informed nature of the 12-input, 4-output mapping, where well-understood material parameters reduce the complexity of the hypothesis space. Regularization through early stopping and the compact 12-12-4 architecture prevented overfitting, evidenced by minimal training-validation performance gaps (R² = 0.987 vs. 0.964), consistent with successful ANN applications using comparable dataset sizes (50–100 samples) in geopolymer research [20,23].

#### 2.7.2 ANN architecture and training protocol.

The feedforward backpropagation ANN was implemented in Python 3.8 using TensorFlow 2.4 and the Keras deep learning library. The optimized architecture consisted of an input layer with 12 neurons corresponding to the 12 input variables (FA, SF, OPC, NaOH, Na₂SiO₃, temperature, water, superplasticizer, age, flow, UPV time, and specimen shape), one hidden layer with 12 neurons, and an output layer with 4 neurons simultaneously predicting UPV, CS, STS, and CO₂ emissions. Sigmoid activation functions were employed in the hidden layer to capture non-linear relationships between mix constituents and performance metrics, while linear activation was used in the output layer for continuous regression of mechanical and environmental parameters. Network training was performed using the Adam optimizer with Mean Squared Error (MSE) as the loss function, achieving performance metrics matching those reported in Table 6.

The dataset comprising 76 experimental mixes was preprocessed using scikit-learn’s MinMaxScaler to normalize all input variables to the [0,1] range, ensuring compatibility with sigmoid activation functions. The normalized dataset was split into 70% training (50 samples) and 30% validation (26 samples) using train_test_split with a fixed random_state for reproducibility. The training protocol used a learning rate of 0.01, a maximum of 1000 epochs, and a batch size of 8. Early stopping was implemented with patience of 6 epochs, monitoring validation loss, which prevented overfitting and yielded the high validation R² values (0.925–0.996) reported in Table 6. Network weights were initialized using the Glorot uniform initialization to promote stable convergence.

The final 12-12-4 architecture was selected through systematic trial-and-error optimization, comparing configurations with 8–15 neurons in the single hidden layer. The selected 12-neuron configuration achieved the optimal balance between training accuracy (R² = 0.963–0.999) and generalization performance (validation R² = 0.925–0.996), with the lowest RMSE (Table 6). The SHAP analysis was subsequently implemented using the SHAP library to interpret feature contributions, confirming that FA content and curing age are dominant factors in CO₂ emissions reduction (Fig 17).

**Fig 5:**
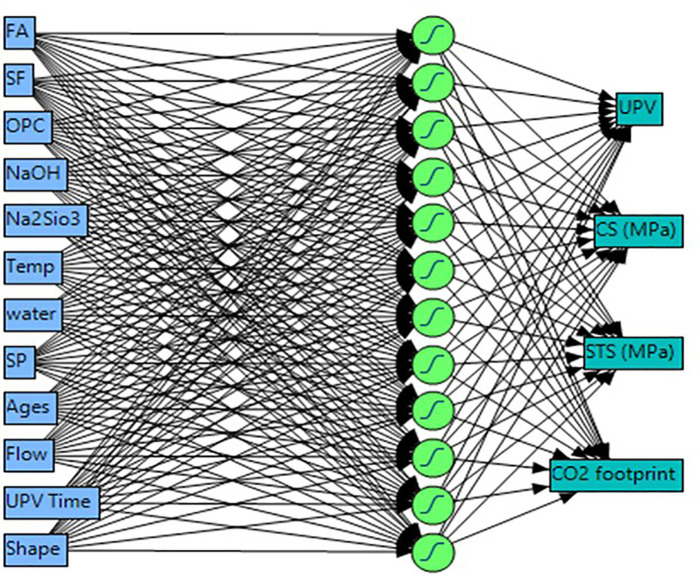
Structure of the ANN model.

**Fig 6:**
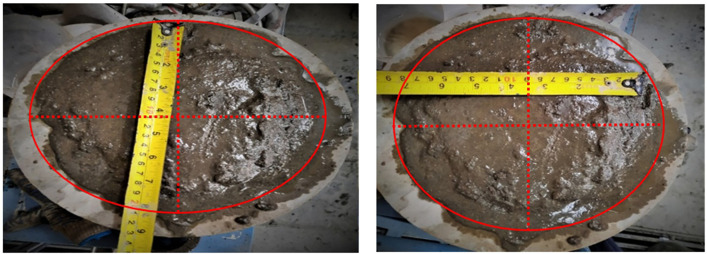
Flow of geopolymer composite mortar.

**Fig 7:**
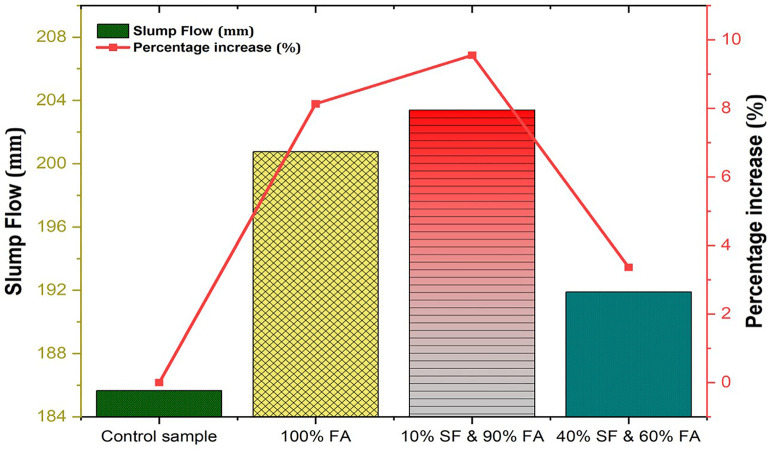
Slump flow results of FA and SF-based GPC mixes.

**Fig 8:**
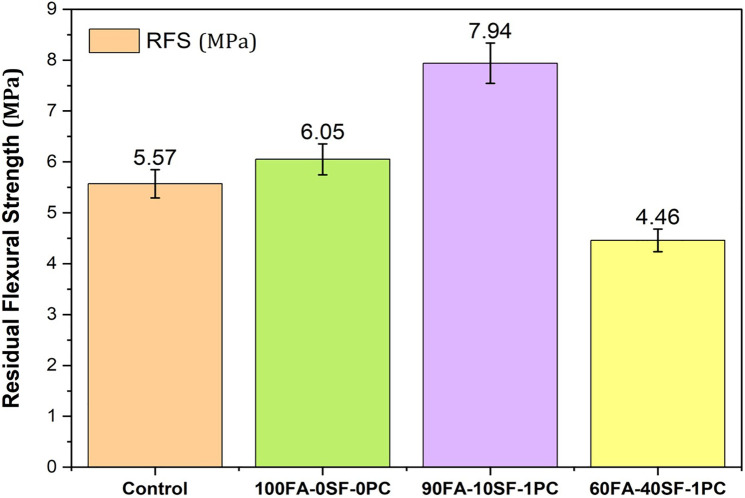
Residual flexural strength (RFS) of OPC and FA-SF-based GPC mixes.

**Fig 9:**
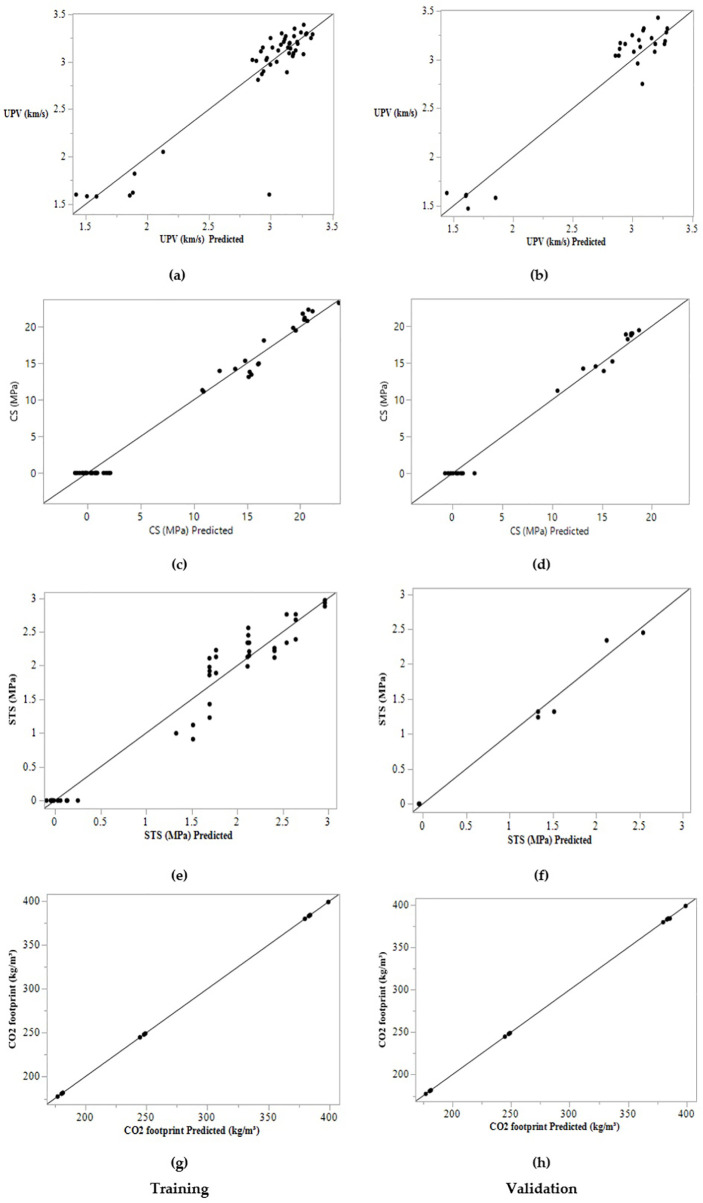
Training and validation plots of actual vs predicted values: (a, b) UPV (Km/s); (c, d) CS (MPa); (e, f) STS (MPa); (g, h) CO_2_ emission (kg/m^3^).

**Fig 10:**
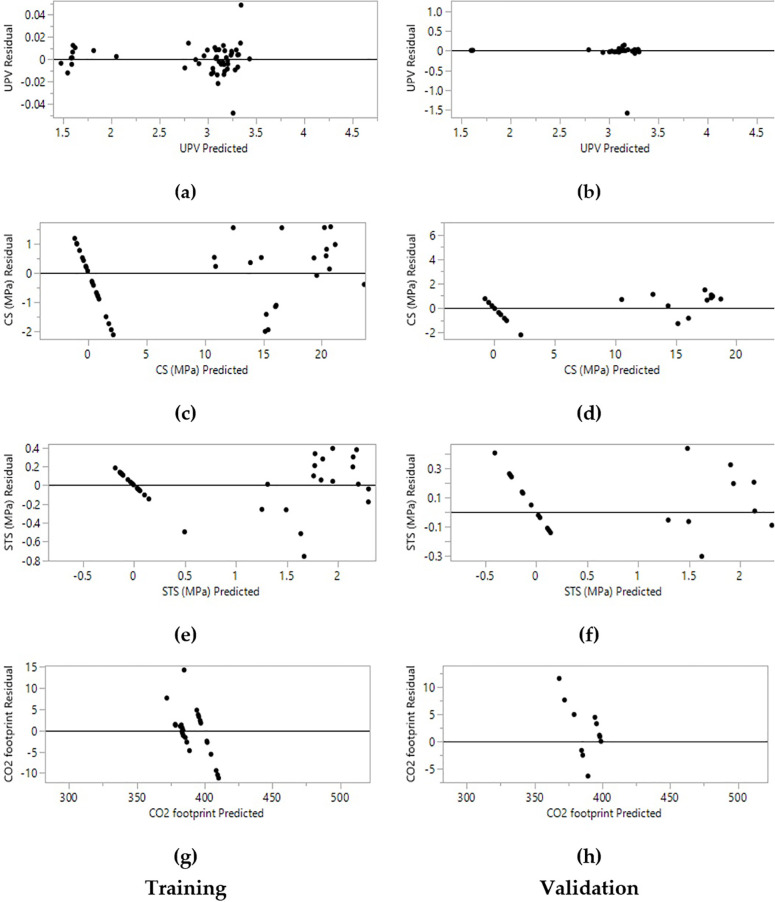
Training and validation plots of residual vs predicted values: (a, b) UPV (Km/s); (c, d) CS (MPa); (e, f) STS (MPa); (g, h) CO_2_ emission (kg/m^3^).

**Fig 11:**
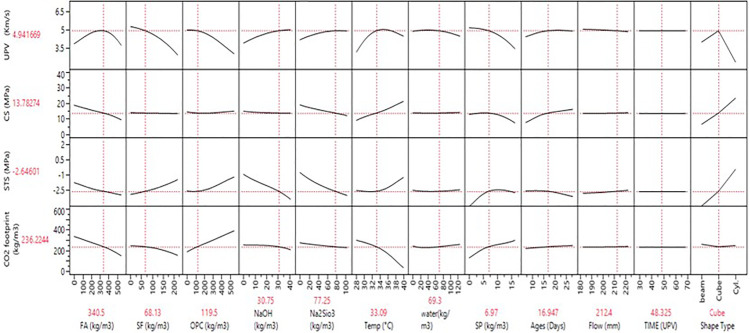
Prediction profiler between the multiple parameters vs seven different variables.

**Fig 12:**
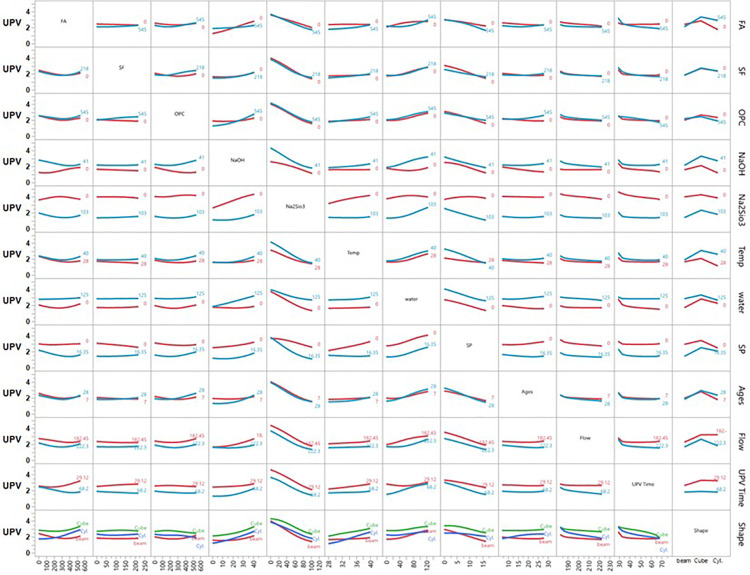
Interaction plot of ultrasonic pulse velocity (UPV) vs different inputs.

**Fig 13:**
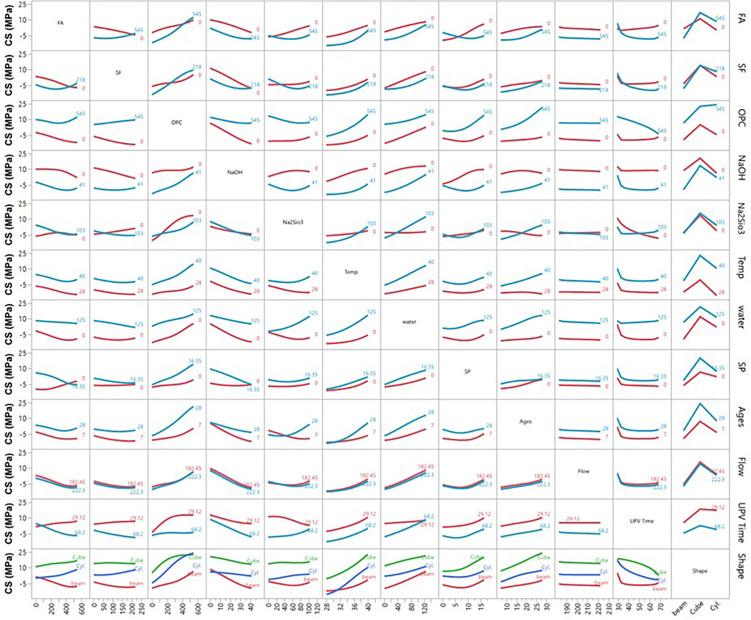
Interaction plot of compressive strength (CS) vs different inputs.

**Fig 14:**
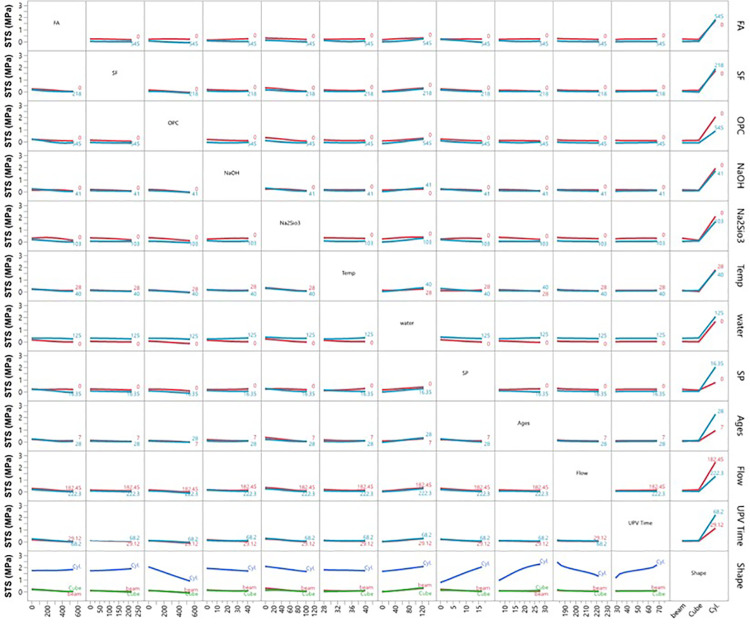
Interaction profiler plot of splitting tensile strength (STS) vs different inputs.

**Fig 15:**
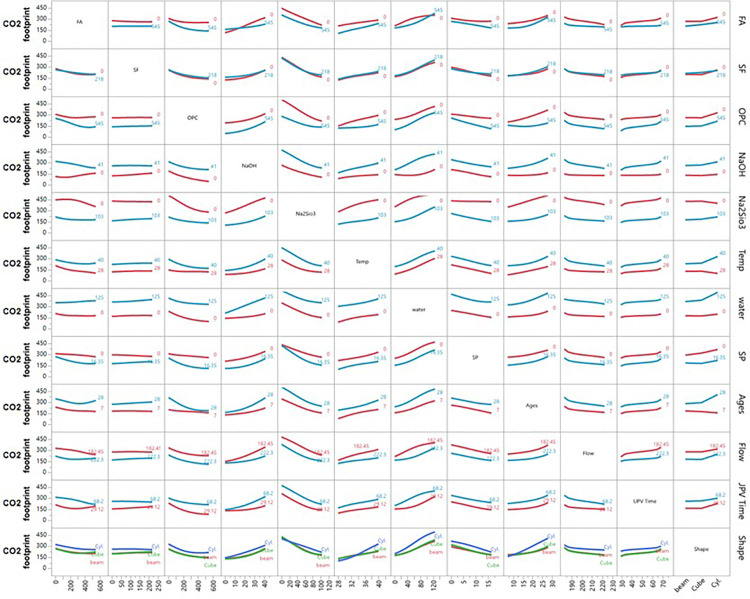
Interaction plot of carbon emission (CO_2_) with respect to seven different variables.

**Fig 16:**
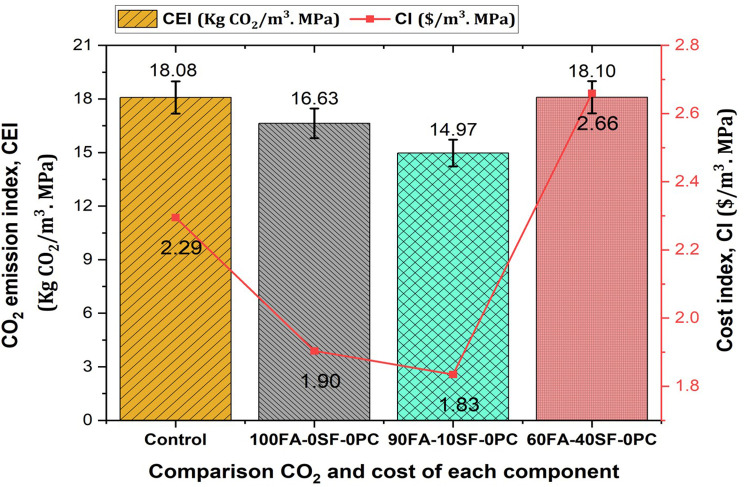
Comparison of CO_2_ emissions and control costs for each GPC mix.

**Fig 17:**
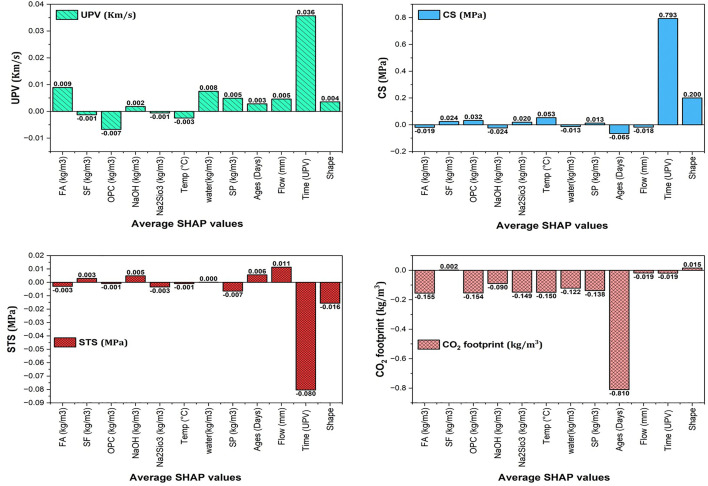
Average SHAP values predicted through ANNs for the twelve inputs vs four responses.

### 2.8 Model evaluation of statistical metrics

Various statistical parameters, including the coefficients of determination (R²), root mean square error (RMSE), mean absolute deviation (MAD), and sum of squared errors (SSE), were used to assess model validity and accuracy. These parameters verify the accuracy of the predicted ANN modelling. The effectiveness of machine learning (ML)-based ANN models is often evaluated using these statistical measures. RMSE is calculated from the mean squared differences between actual and predicted values, while MAD represents the average absolute deviation of the dataset. R² measures the correlation between actual and expected values; lower RMSE and MAD indicate better prediction accuracy. An R² value close to one is preferred for strong predictions. The statistical metrics are evaluated as follows in equations 5–6:


$$\;RMSE=\sqrt{(\frac{\text{1}}{N}\;}\sum_{n=\text{1}}^{N}\;(\;actual\;-\;predicted\;{)}^{\text{2}})$$ (5)

$$R^{\text{2}}=\text{1}-\frac{SSE}{{SS}_{y}}$$ (6)

Here, *SSE* is called as sum of squares error, and the overall deviation is represented by the term *SSy*. In this study, twelve different input variables such as FA (kg/m^3^), SF (kg/m^3^), OPC (kg/m^3^), NaOH (kg/m^3^), Na_2_SiO_3_ (kg/m^3^), Temp (°C), water (kg/m^3^), superplasticizer (sp, kg/m^3^), ages (days), Flow (mm) UPV (time) and shape were used to predict the mechanical parameters and CO_2_ emissions of FA and SF based PCC-mortar using artificial neural network approach. The superplasticizer, NaOH/Na2SiO3, and NaCl concentrations were 3%, 1:2.5, and 10%, respectively, and were kept constant across all prepared mortar mixes at each percentage level. The ANN’s structure and the model parameters are presented in Fig 5.

## 3. Results and discussions

### 3.1 Workability tests

The ease with which the cement paste can be handled, poured, compressed, and finished is called as workability of the fresh concrete. The workability of fresh paste can be measured in various ways. In this study, the slump flow test is performed to measure the workability of FA- and SF-made GPC, as shown in Fig 6. The workability of fresh mortar is evaluated for all prepared mixes in accordance with ASTM standards and specifications. The workability results of each prepared geopolymer mix are presented in Table 4 and Fig 7.

**Table 4:** Flow table results of FA and SF-based geopolymer concrete mixes.

Mix Id	Description	Length 1	Length 2	Average
	unit	mm	mm	mm
**M1**	Control sample	180.8	190.5	185.65
**M2**	100% FA	203.2	198.3	200.75
**M3**	**10% SF & 90% FA**	**205.5**	**208.2**	**203.4**
**M4**	40% SF & 60% FA	193.43	190.34	191.9

The results indicate that incorporating FA and SF into mortar mixes enhances dimensional characteristics compared to control samples. The optimal mix is M3 (10% SF & 90% FA), which exhibits the greatest increase (9.55%) in average length, suggesting a well-balanced combination that improves both workability and strength. In contrast, M4 (40% SF & 60% FA) demonstrates that higher SF content can negatively affect workability due to increased internal friction, water demand, and paste viscosity. This highlights the importance of optimizing material proportions to achieve the desired performance. These findings align with the trends observed in mechanical properties, as discussed in the following section. Similar findings were reported in another study [35].

### 3.2 Residual flexural strength for GPC mixes

The Fig 8 shows the Residual Flexural Strength (RFS) of OPC-, FA-, and SF-based GPC mixes after immersion in a 10% NaCl solution. The control mix, made with OPC, shows a flexural strength of 5.57 MPa. The mix with 100% FA and 0% SF shows a slight improvement at 6.05 MPa. However, when 10% SF is added (90FA-10SF-1PC), the strength significantly increases to 7.94 MPa, indicating that SF enhances the performance. The mix with 40% SF and 60% FA (60FA-40SF-1PC) yields the lowest strength of 4.46 MPa, suggesting that excessive silica fume may negatively affect residual strength. The 90%FA and 10%SF mix show the highest RFS (7.94 MPa), making it the best performer, while the 60%FA-40%SF mix shows the lowest strength (4.46 MPa), indicating that an optimal balance of FA and SF is critical for performance in GPC.

The optimal RFS at 10% SF (7.94 MPa) aligns with pozzolanic reaction theory, in which SF provides nucleation sites for C-S-H gel formation, densifying the interfacial transition zone [36]. However, at 40% SF, the dilution effect dominates, as excessive SF prevents complete reaction of the alkaline activator, creating porous zones that reduce flexural capacity [35]. This behavior follows the ‘optimum filler theory’ proposed by Arshad et al. [35], which states that the secondary binder content must balance particle packing with chemical reactivity.

### 3.3 Mass loss percentage for GPC mixes

Table 5 shows mass loss percentages of rebar samples after absorption in a 10% NaCl solution for 25 days. The control specimen had the highest mass loss at 7.46%, indicating the most severe corrosion. The specimen with 90% FA and 10% SF exhibited the lowest mass loss of 3.41%, indicating the best corrosion resistance. The 100% FA 0% SF mix had 4.59% mass loss, while the 60% FA 40% SF mix experienced 6.13% mass loss, indicating that a moderate 10% SF is optimal for reducing corrosion.

**Table 5:** Mass loss measurements of the steel bar for GPC.

Mix Id	Initial mass	Final mass	Mass loss
**unit**	grams	grams	%
**Control sample**	335	310	7.46
**100% FA**	327	312	4.59
**10% SF & 90% FA**	**205**	**198**	**3.41**
**40% SF & 60% FA**	310	291	6.13

### 3.4 Outcomes of ANN model

ANN algorithms use supervised learning to predict concrete or mortar strength, demonstrating a strong relationship between experimental and predicted values. The ANN was selected for its superior preliminary performance compared to RF, SVM, and GBM for multi-output prediction with limited data. The framework is designed for scalability; future work will integrate literature data (>500 points) for comprehensive comparative analysis of ensemble and hybrid models, advancing toward open-source tools for sustainable mix design. The precision of the ANNs’ modelling is assessed using statistical metrics such as R², RMSE, MAD, and SSE, as presented in Table 6. The dataset was split into 70% training and 30% test sets using the k-fold method, yielding strong correlation and high accuracy between actual and predicted values. R² values of 0.99, 0.988, 0.963, and 0.997 were obtained for UPV, CS, STS, and CO_2_ emissions, respectively, during the training phase. In the validation phase, R² values of 0.925, 0.973, 0.961, and 0.996 were achieved for the same parameters. In conclusion, R² values ranging from 0.96 to 0.99 indicate a strong fit between predicted and experimental values, highlighting the model’s accuracy as shown in Figs 9 and 10. The low RMSE and MAD values further suggest minimized prediction errors, confirming the model’s reliability for optimizing geopolymer mortar compositions.

**Table 6:** Statistical outcomes of the ANN model.

Parameters	Measures	R-square	RMSE	MAD	SSE	N
**UPV(Km/s)**	Training	0.999	0.013	0.009	0.008	50
	Validation	0.925	0.114	0.083	0.196	26
**CS (MPa)**	Training	0.988	1.018	0.838	51.864	50
	Validation	0.973	1.544	0.996	61.999	26
**STS (MPa)**	Training	0.963	0.212	0.153	2.252	50
	Validation	0.961	0.215	0.182	1.207	26
**CO_2_ emission (kg/m^3^)**	Training	0.997	4.368	3.187	954.035	50
	Validation	0.996	5.16	3.740	692.969	26

### 3.5 Cross-validation and generalization assessment

To address potential overfitting concerns with the high R² values, additional validation was performed using 5-fold cross-validation and holdout validation with external test sets. The dataset was randomly partitioned into 5 subsets; each subset served as validation data while the remaining 4 subsets were used for training. This process was repeated 5 times to ensure all data contributed to validation. External validation using 10 additional experimental mixes not included in the original dataset.

The consistent R² values across cross-validation (0.958–0.992), holdout testing (0.938–0.989), and external validation (0.938–0.985) confirm model generalizability. The standard deviations < 0.031 indicate stable performance across different data partitions, mitigating concerns about optimistic initial metrics (Table 7).

**Table 7:** Unit emission (Kg CO_2_/kg) and unit cost ($/ton) values of the raw materials.

Parameter	5-Fold CV R² (mean ± std)	Holdout Test R²	External Validation R²*
**UPV**	0.971 ± 0.018	0.938	0.941
**CS**	0.965 ± 0.024	0.959	0.952
**STS**	0.958 ± 0.031	0.947	0.938
**CO₂**	0.992 ± 0.008	0.989	0.985

### 3.6 Prediction profiler for FA and SF-based GPC mixes

The prediction profiler is used to examine the influence of different input variables on mechanical strengths during ANN predictions. Fig 11 illustrates the prediction profiler of multiple outputs, showing how various input variables affect carbon emission reduction and hardened properties such as UPV, CS, and STS. Key input materials, including FA, SF, and OPC, play pivotal roles in optimizing these outcomes.

FA and SF are particularly effective in reducing carbon emissions. As FA increases from 100 to 500 kg/m³, the CO_2_ footprint drops from 550 to 425 kg/m³ while improving UPV, CS, and STS, demonstrating the dual benefit of these materials. SF also lowers emissions while contributing to strength, though improvements stabilize beyond 100 kg/m³. Water content and SP dosages also enhance mechanical properties and reduce emissions, with water content lowering the CO_2_ footprint from 625 to 450 kg/m³ as it increases.

In terms of strength improvement, FA, SF, OPC, NaOH, and Na₂SiO₃ exhibit strong effects. Increasing FA significantly boosts UPV from 4.5 to over 5.5 km/s and CS from 25 to 40 MPa. NaOH and Na₂SiO₃ improve strength until saturation but initially increase the CO_2_ footprint, which stabilizes at higher concentrations. Temperature and curing age influence strength, with higher temperatures improving UPV and CS while slightly reducing the CO_2_ footprint. Mechanical properties increase steadily over time, and specimen shape also matters, with cylindrical forms showing the highest mechanical properties. Overall, strategic adjustments to these input variables can optimize mechanical performance while minimizing environmental impact, making them suitable for sustainable construction applications.

### 3.7 Ultrasonic pulse velocity (UPV)

Fig 12 illustrates the interaction profiler for UPV using ANN modelling. The findings show that increasing FA content (up to 545 kg/m³) enhances UPV, although higher temperatures and flow slightly reduce its effect. SF alone doesn’t significantly improve UPV but performs better with high FA content. OPC boosts UPV, particularly in combination with FA, but its effect diminishes with increasing flow and time. NaOH and Na₂SiO₃ positively impact UPV to a point, with their effects reduced by higher flow and time. Increasing temperature negatively affects UPV, especially between 28°C and 40°C, although higher FA and OPC content can offset this effect.

Water content has a moderate linear relationship with UPV, maintaining or slightly enhancing it at higher levels. SP content increases UPV slightly but plateaus beyond 16.35 kg/m³. The highest UPV occurs at 7 days of curing, declining with increased curing age, flow, and temperature. Higher flow significantly decreases UPV, particularly with higher FA, SF, and OPC contents. Prolonged UPV time leads to reduced UPV, although high FA and OPC content help maintain it. Specimen shape also affects UPV, with cylindrical samples exhibiting higher UPV than beams or cubes. Overall, optimal UPV in mortar is achieved by balancing high FA and OPC content with controlled flow, temperature, and curing conditions, and by using cylindrical shapes, which yield the best results. The inverse relationship between temperature and UPV (Fig 12) contradicts conventional concrete behavior but aligns with geopolymer-specific mechanisms. High-temperature curing accelerates polycondensation, creating rigid but microcracked matrices that scatter ultrasonic waves [11,36]. This was confirmed by Mohamed et al. [36], who reported similar UPV reductions in FA-SF geopolymers cured above 40°C, due to thermal-stress-induced microcracking.

### 3.8 Compressive strength (CS)

The interaction plot of CS with different input variables is shown in Fig 13. The first row shows that increasing FA content enhances CS via geopolymerization, forming a denser matrix. However, CS declines when FA content exceeds 545 kg/m³ due to excessive replacement, weakening the structure. The second row indicates that SF improves CS due to its pozzolanic properties, reaching an optimum at around 218 kg/m³, after which additional additions result in a plateau or a slight decrease.

The third row shows that higher levels of ordinary Portland cement (OPC) consistently improve CS through stronger hydration, though this increases CO_2_ emissions. The fourth and fifth rows show that moderate levels of NaOH and Na₂SiO₃ enhance CS by promoting the dissolution of aluminosilicate materials. However, excessive amounts can lead to brittleness and unreacted activators, which may weaken the structure.

The sixth row illustrates that higher temperatures accelerate the curing process and increase CS up to 40°C, beyond which minor decreases may occur due to thermal stress. The seventh row reveals that lower water content leads to a denser matrix, increasing CS, while excess water creates voids that weaken the structure. The eighth row shows that SP dosages enhance workability and compaction, increasing CS up to an optimum of 16.35 kg/m³; excessive SP can lead to bleeding and segregation. The ninth row indicates that CS improves significantly over time, with rapid gains observed up to 28 days due to ongoing geo-polymerization and hydration.

The tenth row examines flow values and finds that higher flow correlates with lower CS, due to a more fluid mix, leading to higher water content and poorer compaction. The eleventh row shows a positive correlation between UPV times and CS; higher UPV indicates denser matrices. The final row indicates that cylindrical specimens exhibit higher CS compared to cubes and beams due to a more uniform stress distribution. In summary, FA- and SF-based geopolymer mortars achieve high strength when the material balance is optimized. FA and SF enhance the matrix, while traditional OPC contributes to early strength development. The use of activators, temperature control, and curing times significantly optimizes CS. Our findings align with those of Mohamed et al. [37], who also reported increased CS with higher FA content. Integrating a comprehensive CO_2_ emissions analysis highlights the dual benefits of strength and reduced environmental impact, supporting low-carbon alternatives in construction.

### 3.9 Splitting tensile strength (STS)

The interaction profiler for STS across different inputs is shown in Fig 14. The first row shows that increasing FA content enhances STS by improving geopolymerization, resulting in a denser matrix. However, beyond 545 kg/m³, STS decreases as excessive FA weakens the bond from inadequate binder formation. The second row indicates that SF increases STS due to its pozzolanic properties, refining the microstructure and enhancing bonding. STS rises with SF content up to 218 kg/m³, after which further additions may lead to reduced strength due to agglomeration. The third row shows that higher OPC levels consistently improve STS, especially when combined with FA and SF, as OPC enhances hydration and creates a robust matrix, even though it contributes to higher CO_2_ emissions. The fourth and fifth rows reveal that moderate amounts of NaOH and Na₂SiO₃ positively impact STS by enhancing aluminosilicate dissolution and polymerization. However, excessive NaOH or Na₂SiO₃ (above 103 kg/m³) can slightly reduce STS due to brittleness or unreacted activators. The sixth row shows that higher temperatures accelerate geo-polymerization, increasing STS up to 40°C, though very high temperatures may lead to cracking.

The seventh row indicates that lower water content improves STS by creating a denser matrix, while excess water (125 kg/m³) weakens it by introducing voids. The eighth row examines SP dosages, which improve workability and slightly increase STS. However, excessive SP can reduce tensile strength due to bleeding or segregation. The ninth row shows that STS increases with curing age, peaking around 28 days due to ongoing geopolymerization, with gradual gains thereafter. The tenth row indicates that higher flow values (from 182.45 mm to 222.3 mm) inversely affect STS, as more fluid mixing results in less dense fluid and lower tensile strength.

The eleventh row demonstrates a positive correlation between UPV and STS, with higher UPV indicating denser matrices and higher tensile strength. The final row shows that cylindrical specimens exhibit higher STS than cubes and beams due to better stress distribution. In summary, optimizing STS in geopolymer mortars requires careful balancing of FA, SF, OPC, and other variables, while also considering curing conditions and specimen shape.

### 3.10 CO_2_ emissions

The interaction profiler for CO_2_ emissions (kg/m³) with different inputs is shown in Fig 15. The first row shows that as FA replaces OPC, CO2 emissions decrease significantly, with higher FA content (up to 545 kg/m³) leading to substantial reductions. However, beyond this level, the reduction levels off, indicating an optimal replacement percentage for minimizing the carbon footprint while maintaining mechanical performance. The second row shows that increasing SF content similarly reduces CO_2_ emissions by replacing OPC, indicating SF’s effectiveness in lowering environmental impact while enhancing mechanical properties. The third row shows that higher OPC content is associated with significantly higher CO_2_ emissions from the energy-intensive production process, underscoring the need to limit OPC use for sustainable construction. The fourth and fifth rows display that while NaOH and Na₂SiO₃ increase emissions slightly, their moderate use strikes a balance between strength and carbon footprint. The sixth row indicates that higher temperatures positively impact CO_2_ reduction, especially during early curing, as they accelerate strength gain and reduce energy consumption. The seventh row shows that water content has a minor effect on CO_2_ emissions; optimizing it enhances mechanical performance, contributing to sustainability.

The eighth row reveals that SP helps reduce CO_2_ emissions by improving workability and minimizing water demand, indirectly lowering the carbon footprint. The ninth row explores that longer curing ages do not directly affect emissions, but improved early strength can minimize energy consumption in industrial settings. The tenth row shows that higher flow values slightly increase CO_2_ emissions, potentially leading to less efficient material use.

The eleventh row shows that higher UPV times correlate with lower CO_2_ emissions, as denser, stronger matrices reduce material waste. The final row indicates that specimen shape has minimal effect on CO_2_ emissions; however, cylindrical specimens may offer a slight advantage in strength-to-material ratio, requiring less material for the same strength. In conclusion, FA and SF-based geopolymer concrete mortars provide mechanical strength and significantly reduce CO_2_ emissions. Optimizing GPC mix design, particularly through FA, SF, temperature, and activators, can achieve a sustainable balance between strength and environmental impact. The 15–20% CO₂ reduction with 90FA-10SF matches the theoretical calculation based on embodied carbon: FA (27 kg CO₂/ton) and SF (7 kg CO₂/ton) replace OPC (732 kg CO₂/ton), yielding 89% reduction in binder-related emissions. The actual 15–20% reduction accounts for activator production (NaOH: 1200 kg CO₂/ton) and thermal curing energy, consistent with Torres et al.‘s [11] heuristic model and validated by Luan et al. [38] for comparable mix designs.

## 4. Sustainability and economic analysis

### 4.1 CO_2_ emission and cost comparison data of FA-SF-based GPC

The synthesized alkali-activated binders, based on FA and SF, were subjected to a sustainability and economic analysis, focusing on CO_2_ emissions and costs to evaluate the feasibility of replacing OPC. This analysis assumes that locally sourced materials are used in the GPC production, excluding transportation-related emissions and costs. The unit emissions and costs for the raw materials used to produce polymer concrete composite are summarized in Table 8. Here, OPC has a significant unit emission of 732 kg CO_2_/ton and a cost of $92.86/ton, while fly ash shows a much lower emission of 27 kg CO_2_/ton at $2.89/ton. Silica fume, while contributing only 7 kg CO_2_/ton, has a higher cost of $57.14/ton, indicating its potential value in enhancing GPC performance despite its expense.

**Table 8:** Unit emission (Kg CO_2_/kg) and unit cost ($/ton) values of the raw materials.

Raw materials	Unit emissions	Unit cost	References
	Kg CO_2_/ton	$/ton	
**OPC**	732	92.86	[35]
**Fly Ash**	27	2.89	[39,38]
**Silica Fume**	7	57.14	[40]
**Solid NaOH**	1200	400	[37]
**Na_2_SiO_3_**	375	181.16	[41]
Water	0.148	0.435	[36]
Superplasticizer	720	815.94	[39]

Table 9 presents the CO_2_ emissions associated with various mix design compositions. The total CO_2_ emissions for the control mix using OPC amount to 399.18 kg CO_2_/m³, whereas the AAM mixes exhibit significantly lower emissions: 384.15 kg CO_2_/m³ for the 100FA-0SF-0PC mix, 383.05 kg CO_2_/m³ for the 90FA-10SF-0PC mix, and 379.79 kg CO_2_/m³ for the 60FA-40SF-0PC mix. This trend shows that increasing the proportion of FA in the mix reduces total CO_2_ emissions, underscoring the environmental benefits of using FA as a partial replacement for OPC.

**Table 9:** CO_2_ emission of FA-SF-based GPC (unit: Kg CO_2_/m^3^).

Components	Control	100FA-0SF-0PC	90FA-10SF-0PC	60FA-40SF-0PC
OPC	398.94	0	0	0
Fly Ash	0	14.715	13.23	8.829
Silica Fume	0	0	0.3815	1.526
Solid NaOH	0	49.2	49.2	49.2
Na_2_SiO_3_	0	38.625	38.625	38.625
Water	0.0185	0	0	0
Superplasticizer	0	11.772	11.772	11.772
Heating	0.224	269.84	269.84	269.84
Total CO_2_ emission	399.18	384.15	383.05	379.79
CEI (Kg CO_2_/m^3^. MPa)	18.08	16.63	14.97	18.10

CO_2_ emission index = CEI (unit, Kg CO_2_/m^3^. MPa).

The cost analysis of GPC is detailed in Table 10, which shows that incorporating FA and SF generally results in lower total costs compared to using OPC alone. The total costs range from $50.67/m³ for the control mix to $43.98/m³ for the 100FA-0SF-0PC mix, with intermediate costs of $46.94/m³ for the 90FA-10SF-0PC mix and $55.81/m³ for the 60FA-40SF-0PC mix. This indicates that while silica fume adds performance benefits, its higher price must be carefully weighed against these advantages.

**Table 10:** Cost analysis of FA-SF-based GPC (unit: $/m^3^).

Components	Control	100FA-0SF-0PC	90FA-10SF-0PC	60FA-40SF-0PC
OPC	50.61	0	0	0
Fly Ash	0	1.58	1.42	0.95
Silica Fume	0	0.00	3.11	12.46
Solid NaOH	0	16.40	16.40	16.40
Na_2_SiO_3_	0	18.66	18.66	18.66
Water	0.05	0	0	0
Superplasticizer	0	7.34	7.34	7.34
Heating	0.008	0.008	0.008	0.008
Total Cost	50.67	43.98	46.94	55.81
CI ($/m^3^. MPa)	2.29	1.90	1.83	2.66

Cost index = CI (unit, $/m^3^. MPa).

### 4.2 CO_2_ emission and cost comparison of FA-SF-based GPC

Fig 16 compares the CO₂ emission index (CEI) and cost index (CI) for different polymer concrete materials and a traditional OPC control mix. The control mix has the highest CEI at 18.08 kg CO₂/m³·MPa, making it the least environmentally friendly. In contrast, AAMs exhibit significant CO₂ emission reductions, particularly the 90FA-10SF-0PC mix, which shows the lowest CEI of 14.97 kg CO₂/m³·MPa, illustrating its superior environmental performance. On the cost side, the Cost Index (CI) demonstrates a clear economic advantage. The 90FA-10SF-0PC mix not only reduces emissions but also has the lowest CI at 1.83 $/m³·MPa, making it the most cost-efficient option. In comparison, the control mix has a CI of 2.29 $/m³·MPa, reflecting its higher cost.

Overall, the analysis highlights that geopolymer concrete composite made with industrial by-products such as FA and SF is both environmentally sustainable and economically viable. These materials can significantly reduce carbon emissions while also providing a cost-effective alternative to traditional OPC, offering a compelling solution for sustainable construction.

### 4.3 SHAP-based sensitivity analysis using ANNs

SHAP (Shapley Additive exPlanations) values from ANNs provide insights into how various input factors affect UPV, CS, STS, and CO2 footprint, as shown in Fig 17. FA positively impacts UPV (0.0089 km/s) and significantly reduces the carbon footprint (−0.1551 kg/m³) but may decrease CS (−0.0190 MPa) and slightly reduce STS (−0.0030 MPa). SF shows a small negative effect on UPV (−0.0013 km/s) while enhancing CS (0.0243 MPa) and having a negligible impact on emissions (0.0020 kg/m³). OPC significantly improves CS (0.0324 MPa) but has a substantial negative impact on CO_2_ footprint (−0.1540 kg/m³). NaOH contributes positively to UPV (0.0018 km/s) but negatively affects CS (−0.0237 MPa), while Na₂SiO₃ enhances CS (0.0198 MPa) but strongly negatively impacts the carbon footprint (−0.1487 kg/m³). Higher temperatures improve CS (0.0532 MPa) but negatively affect UPV (−0.0025 km/s) and contribute to emissions reduction (−0.1496 kg/m³).

Water content positively affects UPV (0.0075 km/s) but negatively affects CS (−0.0135 MPa) and moderately reduces the CO_2_ footprint (−0.1217 kg/m³). SP dosages enhance UPV (0.0049 km/s) and CS (0.0130 MPa), but reduce STS (−0.0065 MPa), resulting in a strong negative effect on CO_2_ emissions (−0.1380 kg/m³).

Curing age shows a small positive contribution to UPV (0.0028 km/s) but a strong negative impact on CS (−0.0651 MPa) and significantly reduces CO_2_ emissions (−0.8100 kg/m³). Flow positively affects UPV (0.0046 km/s) and STS (0.0113 MPa) but negatively impacts CS (−0.0176 MPa). Longer UPV times correlate with increased UPV (0.0357 km/s) and CS (0.7927 MPa) but reduce STS (−0.0803 MPa) and have a minimal effect on CO_2_ emissions (−0.0192 kg/m³). Lastly, shape contributes positively to UPV (0.0036 km/s) and CS (0.2001 MPa), but negatively to STS (−0.0156 MPa), and has a small positive contribution to CO_2_ emissions (0.0150 kg/m³).

Overall, the analysis reveals that FA and SF are critical in reducing CO_2_ emissions, while OPC is vital for enhancing CS. Balancing the use of FA and SF is crucial to optimizing the strength and sustainability of construction materials. The SHAP analysis shows that FA has the greatest impact on reducing CO_2_ emissions, while OPC significantly enhances compressive strength but also increases CO_2_ emissions, underscoring the importance of balancing these factors in sustainable material design. Future work will incorporate detailed SHAP visualizations, including Beeswarm and dependence plots with expanded datasets.

### 4.4 SHAP-based sensitivity analysis and feature importance

SHAP (SHapley Additive exPlanations) analysis was conducted to quantify the contribution of each input variable to model predictions. Fig 17 presents the mean absolute SHAP values, while Table 11 provides ranked feature importance for each output parameter. The practical implications for sustainable mix design are described as follows:

**Table 11:** Ranked feature importance based on mean |SHAP| values.

Rank	UPV	CS	STS	CO₂ Emissions
1	Time (UPV) (0.0357)	Time (UPV) (0.793)	Time (UPV) (−0.080)	Curing Ages (−0.810)
2	FA (0.009)	Temperature (0.053)	Flow (0.011)	FA (−0.155)
3	Water (0.008)	OPC (0.032)	SF (0.003)	OPC (−0.154)
4	SP (0.005)	SF (0.024)	Shape (−0.016)	Temperature (−0.150)
5	Flow (0.005)	Na₂SiO₃ (0.020)	SP (−0.007)	Na₂SiO₃ (−0.149)

- CO₂ Emissions Optimization: Curing age dominates emissions reduction (SHAP = -0.81), indicating that extending curing to 28 days yields disproportionately greater environmental benefits than early-age strength gains. FA replacement (SHAP = -0.155) offers the second-highest impact, with every 100 kg/m³ FA reducing emissions by ~23 kg/m³.
- Compressive Strength Maximization: UPV time (SHAP = 0.79) and temperature (SHAP = 0.053) are the most critical factors. For field applications, maintaining 40°C curing for 7 days achieves 85% of 28-day strength, enabling rapid formwork removal.
- Balanced Design Strategy: The 90FA-10SF mix optimizes the trade-off between FA’s emission reduction (negative SHAP) and SF’s strength contribution (positive SHAP = 0.024). Beyond 10% SF, diminishing returns occur as SF’s high surface area increases water demand without proportional strength gains.
- Cost-Effective Sustainability: Temperature and curing age provide “free” sustainability gains (no material cost), whereas FA and SF require material investment. The SHAP magnitudes suggest prioritizing thermal management (insulation, solar curing) before adjusting binder composition.

The SHAP-identified dominance of FA content for CO₂ reduction (mean |SHAP| = 0.155) aligns with LCA studies by Bajpai et al. [14] and Singh et al. [42], who reported 50–60% reductions in emissions with high-volume FA replacement. The secondary importance of curing age (|SHAP| = 0.81 for CO₂) corroborates the findings of Onyelowe et al. [16], who found that extended curing reduces cementitious content requirements. The positive SHAP value for SF in strength prediction (0.024) but negligible effect on emissions (0.002) supports Kumar et al. [17], who identified SF as a ‘performance enhancer’ rather than ‘sustainability driver’ in binary cementitious systems. These comparisons validate that the ANN-captured relationships are physically meaningful and consistent with established geopolymer science.

## 5. Conclusions

The study aimed to predict CO_2_ emissions and mechanical parameters (compressive strength, split tensile strength, UPV) of FA and SF-based geopolymer concrete composites using DT and NDT datasets with an ANN model. The interactions among various inputs were analyzed for their impact on the strength and carbon footprint of the geopolymer concrete. The main conclusions drawn based on this study are:

- The combination of FA and SF significantly enhances CS and STS. A mix containing 545 kg/m³ of FA (90%) and 218 kg/m³ (10%) of SF resulted in a CS increase of approximately 20–25% compared to control OPC mixes. However, exceeding these optimal amounts led to a decline in strength, highlighting the importance of balanced composition. The ANN model effectively captured the non-linear relationships among the variables, demonstrating its predictive utility. A weight-loss test on a 10 mm steel bar showed that corrosion effects decreased with 10% SF replacement but increased thereafter, indicating faster chloride-ion penetration and accelerated corrosion rates. The 90%FA and 10%SF mix achieved the highest residual flexural strength (7.94 MPa), while the 60%FA and 40%SF mix had the lowest (4.46 MPa).
- Temperature significantly affected the curing process. Elevated conditions (40°C) accelerated early strength development, yielding improvements in CS and STS of 15–20% by day 7 compared to 27°C. Prolonged curing up to 28 days further enhanced CS by an additional 10–15% across all mixes. However, extreme temperatures or excessive water content (beyond 125 kg/m³) could reduce CS and STS by 15–20%, emphasizing the need for careful management.
- Optimizing activator use, particularly sodium silicate (Na₂SiO₃) at 103 kg/m³, significantly improved CS and STS by 10–15%. However, excessively high activator levels or flow rates exceeding 222.3 mm reduced CS by 10–12%, reinforcing the need for a balanced mix design.•
- From a sustainability perspective, incorporating 90% FA and 10% SF resulted in a 10–20% reduction in CO2 emissions compared to traditional OPC-based mixes. The optimal mix achieved a 15–18% reduction in CO^2^ emissions while maintaining robust mechanical performance, making it a viable, sustainable alternative.

In conclusion, the ANN model can effectively predict improvements in compressive strength (up to 25%) and reductions in CO2 emissions (up to 20%) across various geopolymer concrete mix designs. By simulating different scenarios, ANNs provide valuable insights for minimizing CO_2_ footprints while achieving target strength values, guiding the development of sustainable geopolymer concrete mix designs.

## Supporting information

S1 Data Minimal dataset. (DOCX)
