# Supplementary material for: Predicting CO2 emissions and strength of FA-SF geopolymer mortar: An ANN approach for sustainable construction
Source: PLoS One. 2026 Apr 3;21(4):e0336654. doi: 10.1371/journal.pone.0336654 (PMC13048446; doi:10.1371/journal.pone.0336654)
Supplement: S1 Data — (DOCX) [file pone.0336654.s001.docx]

Minimal dataset

Chemical composition of SF, FA, and OPC.

| Compound | SF (%) | FA (%) | OPC (%) |
| --- | --- | --- | --- |
| $\mathbf{SiO}_{\mathbf{2}}$ | 91.90 | 55.28 | 15.0 |
| $\mathbf{Al}_{\mathbf{2}}\mathbf{O}_{\mathbf{3}}$ | 0.71 | 26.71 | 2.78 |
| $\mathbf{Fe}_{\mathbf{2}}\mathbf{O}_{\mathbf{3}}$ | 2.54 | 6.65 | 2.72 |
| $\mathbf{CaO}$ | 0.31 | 2.34 | 71.06 |
| $\mathbf{MgO}$ | 1.14 | 0.81 | 1.38 |
| $\mathbf{SO}_{\mathbf{3}}$ | 0.45 | 0.47 | 4.56 |
| ${\text{ }\mathbf{K}}_{\mathbf{2}}\mathbf{O}$ | 1.53 | N/A | 1.21 |
| $\mathbf{TiO}_{\mathbf{2}}$ | 0.01 | 1.89 | N/A |
| $\mathbf{P}_{\mathbf{2}}\mathbf{O}_{\mathbf{5}}$ | 0.63 | 1.92 | N/A |
| $\mathbf{Cl}$ | 0.28 | N/A | 0.08 |
| $\mathbf{MnO}$ | 0.26 | N/A | N/A |

Mix design proportions for FA-SF-based GPC.

| Mix ID | Mix proportion (unit, kg/m^3^) | | | | | | |
| --- | --- | --- | --- | --- | --- | --- | --- |
|  | OPC | FA | Silica Fume | NaOH | Na_2_SiO_3_ | H_2_O | SP |
| Control | 545 | 0 | 0 | 0 | 0 | 125 | 0 |
| 100FA-0SF-0PC | 0 | 545 | 0 | 41 | 103 | 0 | 9 |
| 90FA-10SF-0PC | 0 | 490 | 54.5 | 41 | 103 | 0 | 9 |
| 60FA-40SF-0PC | 0 | 327 | 218 | 41 | 103 | 0 | 9 |
